# Hospital School Teachers’ Sense of Stress and Gratification: An Investigation of the Italian Context

**DOI:** 10.5334/cie.14

**Published:** 2020-03-10

**Authors:** Vincenza Benigno, Chiara Fante

**Affiliations:** 1 ITD, IT

**Keywords:** School at hospital, Teacher job stressors, Teacher job satisfaction, Thematic analyses

## Abstract

In their daily teaching in hospitals, teachers interact within a complex interpersonal and professional network. The present study investigated what kind of professional relationships hospital teachers have with other staff in their daily work and which factors they perceive as being either stressful or gratifying in their professional activities.

An online questionnaire consisting of multiple-choice items and open-ended responses was developed and distributed to all school-in-hospital teachers in Italy. A representative sample of 602 teachers responded. Quantitative findings were analyzed using descriptive statistics. The open-ended responses were analyzed by combining qualitative content analysis with statistical textual analysis using T-LAB software.

The results confirm the complexity of the setting in which hospital teachers operate, one that is characterized by the wide variety of professional and non-professional roles the teachers perform. Four clusters were defined covering both the stress dimensions (Illness, Work Fragmentation, Organization, and Interpersonal Relationships) and the gratifying aspects (Work Recognition, Normalization, Human Contact, and Interpersonal Relationships). The implications of these findings for the management of hospital schools are discussed.

## Introduction

In the current international context, hospital schools are present in most pediatrics departments and are quite well established. In recent decades, many countries have also passed specific laws to regulate their organization, duties, and resources (LeHo Project, 2015).

Hospital teachers operate in very complex settings, where time dedicated to educational activities is necessarily subordinate to children’s treatment needs, and where physical spaces available to the school are often limited and inadequate (Kanizsa & Luciano, 2006; Steike, Elam, Irwin, Sexton, & McGraw, 2016). Moreover, hospitalized students are in a physical and emotional state that is typically non-conducive to learning, and the suffering they and their family members experience requires teachers to take on an emotional support role that is usually not part of educators’ professional training and practice. Poor communication between families, schools, and doctors inevitably has a negative effect on the perception that students and parents have of receiving real support (Kanizsa, 1989).

For these reasons, flexibility a requirement for hospital teachers to function effectively, including instructional planning and choice of learning objectives; synergistic and multidisciplinary work is essential for understanding the hospitalized student’s emotional and educational needs, and to plan effective educational interventions (Capurso & Dennis, 2017). In addition, teachers must work as part of a team, maintain close interaction with families (Asprey & Nasch 2006), collaborate with the student’s mainstream school, and manage any emergencies that may arise (Capurso & Dennis, 2017; Ferraro 2013; Shaw & Brown 2011).

In Italy, all pediatrics departments have a hospital school section with infant, primary, and lower- and upper-secondary school teachers. No specific additional training is provided or required for these teachers, who volunteer to transfer from conventional school to hospital school settings. However, this transition can put the psycho-physical wellbeing of those teachers at risk, given that they receive no support for acquiring the skills needed to operate effectively in such a complex context (Steike et al., 2016).

Mourik (2008) drew up a professional profile of the hospital teacher divided into three dominions, each of which covers a set of specific skills: Dominion A: General Professional Tasks; Dominion B: General Support Tasks; and Dominion C: Specific Support Tasks. Extending Mourik’s work, Capurso and Vecchini (2010) identified six macro-skills areas that a hospital teacher needs to develop: personal/professional, didactic/methodological, organizational, relational/communicative, research-linked, and health-linked.

Despite the differences between hospital schools and conventional educational contexts, few studies have investigated hospital school functioning (Benigno, Fante, & Caruso, 2017; Steike et al., 2016). More specifically, to date, no published study has analyzed stress factors and job satisfaction related to teaching in this specific setting.

Although hospital schools put teachers in an atypical professional condition which, in some respects, may pose risks for their psycho-physical state, they can also represent a particularly rewarding context that acts as a positive catalyst influencing teachers’ attitudes and performance (Caprara, Barbaranelli, Borgogni, & Steca, 2003).

### Purpose of the Present Study

Starting from these considerations, it is possible to hypothesize that the profession of a hospital teacher resembles that of a “helping profession,” often characterized by a strong risk of burnout (Ingersoll, 2001) and subsequent job abandonment (Pithers, 1995). The stress-related and job satisfaction factors, an area widely investigated in the conventional school context (Cooper & Travers 2012; Pearson & Moomaw 2005; Ravichandran & Rajendran 2007; Skaalvik & Skaalvik 2009), represents a first step towards safeguarding the wellbeing of the hospital teacher. That is, the knowledge thus gained can help in activating individual and collective resources and in planning appropriate training courses. Toward that goal, the present study investigated stressors and gratifying factors connected to the professional practice reported by a sample of teachers working in Italian hospital schools.

## Method

The study derives from an investigation into hospital schools conducted in Italy as part of a collaboration between Italy’s Ministry for Education, University and Research (MIUR), the Institute for Educational Technology – Italian Research Council (ITD-CNR) located in Genoa, and Milan Polytechnic’s METID (Methods & Innovative Technologies for Learning) Lab.

A survey disseminated to school-in-hospital teachers was aimed at detecting organizational methods, teaching approaches, use of technologies, training needs, and stressful and gratifying factors connected to the profession of the hospital teacher. Specially, data were gathered and analyzed in order to answer the following research questions:

- What kind of professional relationships do hospital teachers have in their daily work?
- Which aspects of their professional activities do hospital teachers consider stressful or gratifying?

### Participants

The questionnaire was completed by 602 teachers, representing over 90% of the entire population of Italian hospital teachers. Broken down by level, 15.8% taught nursery school/kindergarten, 31.2% primary, 22.9% lower-secondary, and 30.1% upper-secondary school. The sample consisted of 537 females (89.2%) and 65 males (10.8%). The teachers’ age distribution was as follows: 0.8% were between 19 and 29 years old; 7.6% between 30 and 39 years; 24.3% between 40 and 49 years; and 67.3% over 50 years. With regard to experience, 21.9% of the sample had been working in hospital schools for less than two years, 24.9% for 2 to 5 years, 20.6% for 6 to 10 years, and 32.6% for more than 10 years.

### Instrument

A specially designed questionnaire was developed for the study comprised of both multiple-choice items and open-ended questions. The questionnaire is divided into four sections: Personal Profile, Didactic Organization, Use of Technologies, and Professional Dimension. Data collection was carried out by posting the questionnaire online and inviting all teachers in Italy’s hospital schools to complete it (see Appendix A).

#### Relationships

Some items in the Didactic Organization section of the questionnaire were selected to explore the relationships characterizing the work context of teachers in hospitals. For the following questions, respondents were required to specify the purpose of the interaction and its frequency on a 5-point Likert-type scale (1 = never, 2 = once a month, 3 = once every two weeks, 4 = once a week, 5 = when necessary):

- Does your work routine involve systematic relationships with health workers? Indicate the frequency for each type.
- Does your work routine include meetings and interactions with other teaching colleagues in the hospital?

For the following item, also from the Didactic Organization section, respondents were asked to specify the purpose of the interaction and its frequency on a 4-point Likert-type scale (1 = never, 2 = sometimes, 3 = often, 4 = always).

- Does your work routine include meetings and interaction with students’ parents?

#### Stressors and gratifying factors

To investigate stressors and gratifying factors, respondents were asked the following open-ended questions from the section labeled Professional Dimension:

- What stressors are present in your work routine?
- What aspects of your work routine do you consider to be most gratifying?

### Data Analysis

Quantitative findings were analyzed using descriptive statistics (frequency analysis). The teachers’ answers to the open-ended questions were aggregated into two groups, (a) stressors and (b) gratifying factors.

The responses were analyzed in the original Italian language using T-LAB (Lancia, 2004), a program offering a set of linguistic, statistical, and graphic tools for quantitative analysis of texts. Specifically, the Thematic Analysis of the Elementary Contexts (ECs) function was used to construct a thread of discourse within the overall structure of the text. This function is useful for exploring a text for which there is no theoretical basis or empirical evidence, allowing it to be subdivided into subgroups for comparison.

Specifically, the function allows the text to be represented in a few significant thematic clusters that possess the following features: each cluster is made up of “elementary contexts” (sentences, paragraphs) characterized by the same pattern of keywords. Each cluster can be described through the lexical units that are most characteristic of the elementary contexts of which it is made up.

For each cluster, therefore, it is possible to consider the lexical units characterizing it; for each of these the value of χ2 is recorded. Each cluster is briefly described in terms of keywords (lexical units together with the corresponding value of χ2; the threshold value of χ2 for each lemma was 3.84; *p* ≤ 0.05). The researchers identified clusters on the basis of the lemmas and the elementary contexts of which they are made up, as derived from an interpretative process based on their qualitative reading of the texts.

## Results

### Items Related to Relationships Within Work Setting

Tables 1, 2, 3 list respondents’ answers to the following items, respectively:

- Does your work context include systematic relationships with health workers? Indicate the frequency for each type.
- Does your work routine include meetings and interactions with school colleagues in the hospital? Indicate the frequency of each type of interaction.
- In your work routine, meetings with students’ parents are … Indicate the frequency for each type.

**Table 1:** Item “Do you have systematic relations with healthcare staff in your work context?”.

	Never	Once a month	Once every two weeks	Once a week	When necessary

Interactions and meetings with healthcare workers	30.7%	6.3%	1.3%	7.8%	53.8%
Contact sought by staff when they consider it necessary	6.6%	1.5%	0.7%	4%	87.2%
Contact with doctors to ask for information about students	9.5%	1%	0.3%	4.2%	85%
Contact with nurses to ask for information about students	8.6%	0.5%	0.2%	6%	84.7%

**Table 2:** Item “Does your work routine include meetings and interactions with school-in-hospital colleagues? Indicate the frequency of each type of interaction”.

	Never	Once a month	Once every two weeks	Once a week	When necessary

Organization of didactic activity	11.6%	20.8%	21.4%	5.5%	40.7%
Discussion of cases	12.1%	16.4%	18.6%	10.1%	42.7%
Reciprocal support and assistance?	11.6%	12.8%	11.3%	6.5%	57.8%

**Table 3:** Item “In your work routine, meetings with the students’ parents are … Indicate the frequency for each type”.

	Never	Sometimes	Often	Always

Formal, connected to didactic activity	13.5%	27.2%	25.4%	33.9%
Informal, connected to hospital routine	9.1%	21.9%	27.6%	41.4%
Request for support and assistance from families	14.5%	36%	21.3%	28.2%

As illustrated in Table 1, with regard to relationships with medical and healthcare staff, the data suggest that, on the whole, interaction centers on contingencies linked to individual cases; teachers do not participate in formal meetings with the health staff (30.7% stated that they never took part in meetings with the whole team; over 80% declared they contacted or were contacted by the staff “when necessary”).

By contrast (Table 2), respondents reported a high frequency of contacts with hospital school colleagues (“every day”), for didactic reasons (40.7%), for discussion of cases (42.7%), as well as for reciprocal support (57.8%). Only about 10% of participants seemed not to have any cooperative relationships with colleagues (response: “never”).

As shown in Table 3, relationships with families were, on the whole, frequent, and not exclusively centered on the formal aspects connected to didactic activity: 41.4% of respondents noted that they had informal relationships with families, and 28.2% stated that when contacted by families, they “always’ received requests for support.

### Elementary Contexts (ECs)

The results of the thematic analysis of the ECs converged around four thematic clusters of both stressor and gratification factors. Each cluster is described in terms of “typical words” (lexical units with the corresponding χ2 value), and assigned a label based on qualitative interpretation performed by analyzing the ECs grouped in each theme as well as the words connected to each cluster.

#### Stressors

The corpus of answers on stress factors given by the hospital teachers consisted of 7,828 occurrences (single lemmas). The four clusters identified through the Thematic Analysis of the Elementary Contexts function accounted for 29.15%, 15.79%, 37.65%, and 17.41% of the variance, respectively. The clusters along with some sample words are shown in Table 4. The four clusters related to stress factors were interpreted as follows.

**Table 4:** Reported Stressors, Clusters, and Characteristic Lemmas.

Characteristic lemmas	χ2 value^1^	Characteristic lemmas	χ2 value

**Contact with illness** 29.15%		**Organizational problems** 37.65%	
contact	68.61	didactic	22.27
pain	44.14	activity	20.31
suffering	36.25	appropriate	16.27
factor/cause	18.85	personal	13.39
stressful	21.29	educational	12.45
emotional	17.88	space	10.70
death	15.92	unavailability	10.27
physical	14.17	interrupt	9.90
constant	13.75	technological	7.70
involvement/relation	11.99		
**Work fragmentation** 15.79%		**Intensive relationships** 17.41%	
time	34.10	relate	32.43
timetable	32.93	impact	21.61
numerous	25.56	role	21.61
need	22.91	intensive	20.88
patient	20.15	build	15.72
necessary	20.03	difficult	10.90
meet	17.60	attempt	10.70
morning	17.60	positivity	10.70
ward	7.90	parent	7.25
		relation	7.18
		family	5.75

##### Cluster 1: Contact with illness

The first cluster indicates that, for many teachers, exposure to the suffering of the students and their families constituted a stressor, as did management of their own and others’ emotional reactions. This cluster represents student and family reactions to illness: the teachers constantly found themselves having to deal with others’ experience of pain and sometimes death, aspects that are not a part of their training or a characteristic of conventional professional activity. Typical examples of this EC included:

**Contact** with chronic **illnesses** (…) empathizing with the young patients’ **pain** (…) **constant contact** with the students’ and their families’ **suffering** and pain (…) **continuous contact** with illness, suffering, death (…) managing **emotional relations** with those experiencing pain (…).

##### Cluster 2: Work fragmentation

This cluster is linked to the work context, which is regarded as a critical aspect of the teachers’ professional activity, particularly organization of work. Two elements considered by many participants as a source of stress were the difficulty of delivering lessons without continual interruptions and the brevity of lessons imposed by context and time management constraints. Typical examples of this EC were as follows:

The organization of time when students can’t move out of their rooms and are particularly **numerous** (…) the extreme flexibility of lesson **times** due to treatment requirements (…) the number of **patients** that continuously varies (…) each student has a different **need**, and the lesson is often interrupted by the various therapies (…) short **times** for didactic activity and frequent interruptions.

##### Cluster 3: Organizational problems

The third identifying factor covers organizational aspects of the work context, such as spaces that are inappropriate and not equipped for school work, the need for a continuous didactic reorganization, and the need for sudden adjustments in response to the heterogeneous nature of the student population. Other challenges included the need to maintain contacts and an ongoing relationship with the students’ schools of origin, as well as difficulties connected to management of the spaces of the school in hospital. Typical examples of this EC were as follows:

Difficulty holding lessons in the presence of serious diseases (…) Difficulties linked to continual adaptation to the syllabi of different study paths and classes (…) Lack of a place **appropriate** for school purposes (…) Lack of **technological** tools and **learning** materials (…) Lack of a **place** specifically structured for school purposes (…).

##### Cluster 4: Intensive relationships

The fourth factor includes relational aspects linked to the context in which the teachers found themselves working. Greater interaction is required of them than in conventional schools in terms of the number and diversity of “actors” involved (medical staff, families, colleagues both in the hospital school and in students’ school of origin) and also in terms of the “simultaneity” of the contacts to be managed. Relationship difficulties also seem to involve organizational and logistic aspects. Often, not having a suitable space specifically devoted to school activities led to greater contact with the other figures present in the ward, such as colleagues, families, and healthcare workers. Typical examples of this EC were as follows:

Being in a **relationship** with children and their **parents** who experience **intensive** emotions and at the same time being able to maintain control and support them (…) The initial **impact** with the emotional experience of parents (…) the daily effort of the **family** related to the child’s illness (…) Sometimes when the parent interferes in the child’s work (…) Sometimes misunderstandings when relating with others in the ward (…)

#### Gratifying factors

The corpus of answers given by the teachers regarding the gratifying factors of their working practice consisted of 2,342 occurrences.

The four clusters identified through the Thematic Analysis of the Elementary Contexts function accounted for 36.24%, 25.84%, 22. 82%, and 15.10% of the variance, respectively. The typical words (lexical units and corresponding χ2 value) characterizing each cluster are shown below in Table 5. The four clusters related to gratifying dimensions were interpreted as follows.

**Table 5:** Reported Gratifying Factors, Clusters, and Characteristic Lemmas.

Characteristic lemmas	χ2 value	Characteristic lemmas	χ2 value

**Work Recognition** 36.24%		**Human Contact** 22. 82%	
work	41.22	smile	30.81
carry out/do	31.22	human	16.68
return	23.40	strong	14.88
see	18.85	sharing	11.90
hospital	15.94	situation	11.90
anxiety	15.94	normality	11.22
find	13.63	student	10.36
teacher	10.24	boy/girl	10.24
leave	9.73	learner	7.98
**Normalization** 25.84%		**Interpersonal Relationships** 15.10%	
hospitalization	31.17	set up (verb)	54.14
path	31.17	wait	26.50
alleviate	27.91	relative (adj.)	26.50
distress (n.)	23.11	interaction	23.81
suffering	22.29	different	23.55
educational	20.10	relations	23.10
health (adj.)	16.58	immediate	13.50
contribute	12.95	personal	10.33
problems	12.22	importance	8.11
collaboration	12.05	request	8.11
effective	11.35		

##### Cluster 1: Work recognition

The teachers identified external recognition of the work they had done as a gratifying element of their professional practice – recognition not only from students and parents, but also from medical teams and from their colleagues. Synergic teamwork emerged as a further possible source of job satisfaction. Typical examples of this EC were as follows:

When parents come back to see me in **hospital** to thank me for the **work** done with their son/daughter (…) Teamwork with the other workers on the ward (…) The thanks often expressed by the doctors for the help the teachers give with their competence (…) Positive and constructive relationship with colleagues and other hospital workers (…).

##### Cluster 2: Normalization

Another element that the teachers found gratifying was the possibility that the work they carried out in the hospital context might give the students and their families a sense of continuity to their normal lives. Thus, this cluster is represented by the significance that, according to the respondents, didactic activity takes on at **this particular moment** in the students’ and their families’ lives. In the hospital context, schooling seems to play a special role, that of restoring continuity to the lives of the children/young people, who are experiencing a sense of interruption caused by their illness and hospitalization. Moreover, the teachers maintained that didactic activity can reduce and alleviate the condition of distress and suffering, and this aspect was perceived as a gratifying dimension of their profession. Typical examples of this EC were as follows:

Alleviating the distress of **hospitalization** (…) Realizing that the didactic activities are an important opportunity to escape from the problems connected to hospitalization (…) Awareness that our work **alleviates** the **distress** deriving from hospitalization (…) The children’s chance to recuperate everyday normality.

##### Cluster 3: Human contact

Direct contact with the students and their families, and the gratitude they express, were recognized as gratifying factors. Typical examples of this EC are as follows:

Human contact with the children, and their **smiles** (…) Representing the **human** aspect and contact with everyday life for pupils and parents (…) The enthusiasm and concentration with which the **students** participate in the activities proposed by the school (…) The special smile of our children, and the parents’ trust in the teachers, which is a special kind of **sharing**.

##### Cluster 4: Interpersonal relationships

The chance to establish significant relationships in their work context was considered by respondents as an important element of gratification. Typical examples of this EC were as follows:

**Establishing** significant relationships with the children (…) If a trusting **relationship** is established, progress is evident (…) The **relationship** with the children and their families (…) Success in the **relationship** with the pupil (…) **Establishing** trusting relationships with the users (…) The relationship with the students and medical staff.

The third and fourth clusters involve gratification connected to the context of work relationships. That is, the teachers perceived the human aspect in the relationships they establish as gratifying, particularly the possibility of serving as educational figures who are able to restore positive emotions in a context that is characterized by physical and emotional pain. The enthusiasm and concentration with which the students participate in the activities the school proposes was considered to be a strongly gratifying element. Finally, the building of relationships defined as “significant,” centered on trust, was another element of potential satisfaction connected to respondents’ working practice.

## Discussion

The teacher-in-hospital profession is characterized by unique contextual elements that require teachers to demonstrate a considerable capacity for flexibility in instructional planning and organization of their work, as well as good interpersonal and communication skills.

The findings of the survey reported on here confirm the complexity of the relational setting in which the hospital teacher operates. As Steinke et al. (2016) highlighted, hospital teachers’ communication with onsite medical and psychosocial staff is an essential component of their daily schedule. This setting is moreover characterized by an alternation of formal and informal contacts, and also by a wide range of professional and non-professional roles performed by the teachers involved. Indeed, the findings show that teachers find themselves having to interact very frequently with the medical team, mostly in unpredictable contingencies that emerge in the management and organization of their work.

Moreover, contacts with students’ parents are much more frequent and very different from those in traditional school practice, since they are centered in part on aspects that do not directly involve didactic and/or social-related activity, being frequency focused on offering families support.

Stressors deriving from contact with students’ and their families’ physical and psychological pain can be considered specific to the work of teachers in the hospital context. This often requires hospital teachers to be particularly resilient and to employ coping strategies that tend to be more characteristic of healthcare professions **(*Contact with illness*).**

The other dimensions that emerged from the analysis; namely, issues connected with the fragmentary nature of lessons and difficulties in organizing times and spaces **(*Work fragmentation and organizational problems*)** also seem mostly to be environmental features typical of the work context of hospital teachers. These may have a notable effect on hospital teachers’ sense of professional self-efficacy. Although organizational elements have been recognized as variables that may affect the perception of work-related stress in school teachers generally (Ravichandran & Rajendran, 2007), in the hospital context specific factors may play an important role, since the “school environment” is subordinate to treatment routines.

As mentioned, the findings of the survey suggest a high frequency of contacts among colleagues, an element that reduces the impact of stress on teachers; relations with the medical team and healthcare workers seem mostly to center on specific and contingent needs rather than occurring within formally organized meetings and exchanges. The lack of formal integration of the function of the teachers into the hospital context may negatively influence their perception of their work reality and role, and may trigger specific relational difficulties. Thus, the lack of meetings with the healthcare team and lack of recognition of their work within the hospital context may result in teachers “feeling like strangers whose presence is tolerated” (Kanizsa, 1989 p.47; ***Intensive relationships***).

As for the gratifying factors that emerged, external recognition of one’s work by all the staff involved in the context may be considered as an extrinsic motivating factor that makes the profession of hospital teachers similar to that of “conventional” teachers **(*Work Recognition*)**. However, in the case of hospital schools, the teacher comes into contact with professional staff who are normally extraneous to their working context, such as doctors and healthcare workers.

In addition, the findings show the extent to which synergic group work can be considered as a gratifying factor compared to isolated, independent work. It is possible that factors like the fragmentary nature of lessons, the inevitable challenges in organizing content, spaces and times, as well as emotional stress all cause hospital teachers to seek more support and team work, thus calling for a more cooperative approach to their activities.

Giving meaning during a critical moment in students’ lives and potentially contributing to the maintenance of a condition of normality oriented towards the future and future plans seem to be important elements of gratification reported by the respondents **(*Normalization*)**.

The relational dimension was recognized by respondents as an important element of gratification **(*Human Contact* and *Interpersonal relationships*).** In the hospital context, the ability to prepare a sound teaching program is seen to be secondary to the creation of solid, trust-based relationships with students and their families (Benigno et al., 2017). As such, Reyhani, Aemmi, & Zeydl (2016) reported that the presence of a teacher at the child’s bedside is a simple and effective strategy to reduce mothers’ anxiety regarding their child’s hospitalization.

The satisfaction in their professional activities reported by the hospital teachers seems partly to be connected to elements that are intrinsic (Caprara, Barbarabelli, Steca, & Malone, 2006) to the profession and that, in some ways, make it similar to the “caring professions.” According to Kanizsa (1989), the motivation to pursue a caring vocation within teaching is one of the elements that motivate teachers in Italy to teach in hospital schools.

## Conclusion

Hospital schools may be considered a special kind of boarding school, where teachers need to recognize and address the specific factors of the hospital as a care-giving setting. Some elements of hospital schools can become a source of stress for teachers; at the same time, despite the disruptive experience that hospitalization represents for young patients, providing them with a path of normality and continuity through schooling can be a source of gratification in teachers’ professional activities.

The findings presented here have important implications at both the personal and the organizational/institutional level. On a personal level for hospital school teachers, the study is a first step towards identifying and analyzing both the stressors and the gratifying factors that characterize their work, which is only partly comparable to that of teachers in conventional school settings (Cooper & Travers, 2012; Kyriacou, 2001; Pearson & Moomaw, 2005; Ravichandran & Rajendran, 2007; Skaalvik & Skaalvik, 2009). Some variables related to the hospital context are clearly a source of stress for the teacher. Furthermore, these factors must be adequately managed to prevent stress-related disorders and burnout.

From an institutional point of view, careful analysis of the variables identified in this study may help to identify a set of basic skills required for this professional category, possibly leading to the provision of appropriate training. In particular, this skill set should include management of intense emotional experiences and relationships characterized by suffering.

The teaching of communication skills (assertiveness and social skills training) and functional coping strategies (emotion-focused strategies, problem-focused strategies, and coping skills training) could also prove particularly effective in relation to the hospital context. Moreover, it is clear from an analysis of the data that hospital teachers need specific training in how to develop innovative and flexible teaching strategies and activities that adapt to the particular educational needs of hospitalized students and to the time constraints of the hospital school. Furthermore, the findings from this study can help to refine the professional profile of hospital teachers identified in other studies (Capurso & Vecchini, 2010; Mourik 2008).

The gratifying factors identified in the study might contribute to and support hospital teachers’ sense of self-efficacy, counteracting the typical sense of impotence connected with experiencing student and family suffering. Indeed, teachers’ self-efficacy, in general, is strongly driven by their sense of having positively influenced students’ academic results and fostered their overall development. In the case of hospital schools, additionally, teachers may gain satisfaction from maintaining hospitalized students’ motivation, as well as promoting their interest in following and engaging with the outside world, despite the suffering they are experiencing.

Moreover, analysis of the gratifying factors shows that the teachers consider recognition of their work and the importance of the school in hospital, both by the family and by social and healthcare workers, as important positive elements. Therefore, a significant step that could be taken at the institutional level would be to help integrate/assimilate the teacher within the team operating in the hospital context. Without such measures, the relative isolation of hospital teachers may negatively influence their perception of their work situation and role, besides leading to specific relational difficulties.

The authors’ investigations of hospital schooling in Italy have provided the basis for developing an integrated model for hospital teachers’ professional growth (Benigno, Fante, Epifania, Caruso, & Ravicchio, 2018). This model encompasses two major thematic areas: (a) psycho-relational aspects connected with student illness and teacher stress management; and (b) methodological aspects regarding the design and implementation of learning paths specifically for hospital schools.

The study’s limitations are largely related to the methodology used. Despite the use of a quantitative analysis tool, the data consist of self-reported responses to open-ended questions. This methodological approach seemed useful for performing a preliminary explorative analysis of the constructs to be studied. Indeed, the findings that have emerged might help future efforts to develop quantitative tools that are more specific to the variables related to this particular professional category.

Another limitation involves the subjects of the study. That is, we examined exclusively teachers working in Italy. Consequently, our findings might not be generally applicable to hospital schooling in other countries. Nevertheless, we hope that the variables considered will prove useful for future study in other countries.

Finally, the research undertaken here represents a first step in analyzing hospital teachers’ stress and gratifying factors. It is recommended that future studies investigate which intrinsic factors (personality traits, coping style, locus of control) and which extrinsic factors (job definition, administrative, organizational, and didactic aspects of the hospital school) affect the relationship between stressors and teacher responses.

## Additional File

The additional file for this article can be found as follows:

10.5334/cie.14.s1 Appendix 1. Teachers’ questionnaire on School in Hospital.

## Footnotes

Threshold value of χ2 for each lemma was 3.84; *p* ≤ 0.05.
