# Supplementary material for: Hospital School Teachers’ Sense of Stress and Gratification: An Investigation of the Italian Context
Source: Contin Educ. 2020 Mar 10;1(1):37–47. doi: 10.5334/cie.14 (PMC11104412; doi:10.5334/cie.14)
Supplement: Appendix 1. — Teachers’ questionnaire on School in Hospital. [file cie-1-1-14-s1.pdf]

Appendix 1

Teachers’ questionnaire on School in Hospital

Dear Teacher,

We invite you to complete this questionnaire , as part of our efforts to collect information on your educational activity with hospitalized students.

We would like to know more about the work of hospital teachers like yourself, especially the main organizational and didactic methods, the habitual use of technologies, professional learning needs, the stressor and gratifying factors, and how the School in Hospital Portal (PSO) service is used.

The information you provide will be useful for calibrating new services for the permanent training of hospital teachers, which will become available in the future through the PSO portal.

It will take no more than 15-20 minutes to complete the questionnaire.

Your answers will be treated with the utmost confidentiality and will be used exclusively for the planning phase of the initial teacher training activity. In no way will they be used for evaluation purposes.

We thank you in advance for your kind cooperation.

PERSONAL PROFILE

Gender

- Female
- Male

Age

- 10-29 years old
- 30-39 years old
- 40-49 years old
- 50 years or over

Education

- High School graduate
- Bachelor's degree
- Master's degree
- Doctorate degree

School level

- Pre-primary
- Primary
- Lower Secondary
- Upper Secondary

Please indicate in which hospital ward you work.

How long have you been working as a teacher at the hospital?

- less than 2 years
- 2 - 5 years
- 6-10 years
- more than 10 years

DIDACTIC ORGANIZATION

Please indicate the location/setting where you mainly carry out your teaching activity in the hospital.

- Day Hospital
- A single ward
- Multiple wards
- Other

Do you have regular and systematic contact with medical/healthcare staff? Indicate the frequency for each type.

	Never	Once a month	Once every two weeks	Once a week	When necessary
I am included in and attend meetings with the healthcare team	○	○	○	○	○
I am in contact with the medical staff when they consider it appropriate	○	○	○	○	○
I contact the doctors to ask for information and support about my students	○	○	○	○	○
I contact the nurses to ask for information and support about my students	○	○	○	○	○

In the hospital where you work, is there a space dedicated to school activities? *

- No
- Yes
- There is a space I can use, but it is not exclusively used for educational activities
- Other

Are you in contact with the hospitalized students' school of origin?

- Yes
- No

If so, which mode do you use and how often?

	Never	Once a month	Once a week	Two or three times a week	Every day
Email	○	○	○	○	○

	Never	Once a month	Once a week	Two or three times a week	Every day
Phone	○	○	○	○	○
Online class register	○	○	○	○	○

Do you plan activities in collaboration with the class of origin?

- Yes
- No

If so, please indicate which activities and how often they are carried out.

	Never	Once a month	Once a week	Two or three times a week	Every day
Maintain contact with peers	○	○	○	○	○
Implement educational activities together with the origin classroom	○	○	○	○	○

What teaching strategies do you adopt?

	Never	Once a month	Once a week	Two or three times a week	Every day
Lecture	○	○	○	○	○
Activities in a small groups	○	○	○	○	○
Brainstorming	○	○	○	○	○
Drill&pratice	○	○	○	○	○
Project-based learning	○	○	○	○	○
Game-based learning	○	○	○	○	○

Do you have opportunities to meet up and interact with school colleagues in the hospital for:

	Never	Once a month	Once a week	Two or three times a week	Every day
Organization of educational activities	○	○	○	○	○
Discussion about a student progress	○	○	○	○	○
Reciprocal support	○	○	○	○	○

What kind of relationship do you have with your students' parents?

	Never	Sometimes	Often	Always
Formal, related to educational activity	○	○	○	○
Informal, connected to the hospital routine	○	○	○	○
Providing help and support	○	○	○	○

TECHNOLOGY USE

How often do you use the following applications for preparing and carrying out your lessons?

For each item, select the frequency 0 (never), 1 (once a month), 2 (once a week), 3 (two or three times a week), 4 (every day)

	Computer	Mobile device (e.g.Tablet)
Word processor (e.g. Microsoft Word)		
Spreadsheet (e.g. Microsoft Excel)		
Presentation Software (e.g. Microsoft PowerPoint)		
Graphic Editor		
E-mail		
Online Forum		
Social networks		
Videoconferencing		

	**Computer**	**Mobile device (e.g.Tablet)**
(e.g. Skype)		
File sharing systems (e.g. Dropbox)		
Collaborative writing environments (e.g. wikis)		
Learning Management Systems (LMS)<br>(e.g. Moodle)		
Educational Software		
Apps		

In what other ways, if any, do you use digital technologies during lessons?

How often do your hospitalized students use the following applications and digital tools for learning?

For each item, select the frequency 0 (Never), 1 (once a month), 2 (once a week), 3 (two or three times a week), 4 (every day)

	**Computer**	**Mobile Device (e.g. Tablet)**
One or more of the most widespread applications (word processors, spreadsheets, etc.)		
Environments for interpersonal communication (E-mail, forums, Social networking, etc)		
Videoconference (e.g. Skype)		
Sharing environments (e.g. Dropbox)		
Collaborative writing environments (e.g. wikis)		
Educational Software		
Apps		

OPEN SURVEY QUESTIONS: PROFESSIONAL DIMENSION

- **What stressors do you experience in your work routine?**

- **What aspects of your work routine do you consider to be most gratifying?**

- **What are your professional learning needs?**
